# Neurocognitive Development of the Resolution of Selective Visuo-Spatial Attention: Functional MRI Evidence From Object Tracking

**DOI:** 10.3389/fpsyg.2018.01106

**Published:** 2018-07-30

**Authors:** Kerstin Wolf, Elena Galeano Weber, Jasper J. F. van den Bosch, Steffen Volz, Ulrike Nöth, Ralf Deichmann, Marcus J. Naumer, Till Pfeiffer, Christian J. Fiebach

**Affiliations:** ^1^Department of Psychology, Goethe University Frankfurt, Frankfurt am Main, Germany; ^2^Institute of Psychology, University of Education Karlsruhe, Karlsruhe, Germany; ^3^IDeA Center for Individual Development and Adaptive Education, Frankfurt am Main, Germany; ^4^Institute of Medical Psychology, Goethe University Frankfurt, Frankfurt am Main, Germany; ^5^Brain Imaging Center, Goethe University Frankfurt, Frankfurt am Main, Germany

**Keywords:** selective attention, visuo-spatial attention, object tracking, development, functional magnetic resonance imaging (fMRI)

## Abstract

Our ability to select relevant information from the environment is limited by the resolution of attention – i.e., the minimum size of the region that can be selected. Neural mechanisms that underlie this limit and its development are not yet understood. Functional magnetic resonance imaging (fMRI) was performed during an object tracking task in 7- and 11-year-old children, and in young adults. Object tracking activated canonical fronto-parietal attention systems and motion-sensitive area MT in children as young as 7 years. Object tracking performance improved with age, together with stronger recruitment of parietal attention areas and a shift from low-level to higher-level visual areas. Increasing the required resolution of spatial attention – which was implemented by varying the distance between target and distractors in the object tracking task – led to activation increases in fronto-insular cortex, medial frontal cortex including anterior cingulate cortex (ACC) and supplementary motor area, superior colliculi, and thalamus. This core circuitry for attentional precision was recruited by all age groups, but ACC showed an age-related activation reduction. Our results suggest that age-related improvements in selective visual attention and in the resolution of attention are characterized by an increased use of more functionally specialized brain regions during the course of development.

## Introduction

Visual selective attention is the ability to modulate perception in order to optimize processing according to our current task goals (Carrasco, 2011). We can apply visual selective attention rather coarsely or we can scrutinize our environment with a fine grain – limited by what has been termed the *resolution of attention* (He and Cavanagh, 1996). The resolution of attention is the minimum size of the focus of attention, i.e., of the region that can be selected by attention, and is as small as 3–5 min of arc in foveal vision in adults (Toet and Levi, 1992; Intriligator and Cavanagh, 2001).

Developmental research suggests that several aspects of children’s selective attention undergo substantial changes in early childhood (e.g., Ridderinkhof and van der Stelt, 2000; Scerif, 2010), including the resolution of attention (Wolf and Pfeiffer, 2014). The ability to constrict the focus of attention to increasingly smaller sizes changes markedly between 7 years of age and adulthood; for 9- to 13-year-old children and adolescents, it is about twice the size as for adults and for 7-year-olds three times the size as for adults (Wolf and Pfeiffer, 2014). Similar findings have been reported by studies using flanker (Enns and Girgus, 1985; Pastò and Burack, 1997) and crowding paradigms (Bondarko and Semenov, 2005; Jeon et al., 2010; Huurneman et al., 2014; Norgett and Siderov, 2014). However, the neural mechanisms underlying the development of attentional resolution are at present unknown. This problem touches upon two intimately related aspects, namely the development of the neurocognitive mechanisms underlying visuo-spatial selective attention *per se* and the specific development of the precision of visuo-spatial selective attention. Developmental research on both topics is so far scarce.

Regarding the neural basis of visual selective attention, adult and primate research has converged on the view that selective attention can be conceived as a modulation of perceptual processes, with modulatory signals originating from a fronto-parietal network comprising frontal eye fields (FEF), posterior parietal cortex (PPC, most specifically the superior parietal lobule/SPL and intraparietal sulcus/IPS), and the superior colliculi, and with sensory areas as targets of these modulatory signals (for reviews see Baluch and Itti, 2011; Moore and Zirnsak, 2017).

Despite obvious developmental changes in the ability to voluntarily deploy attention (Scerif, 2010; Amso and Scerif, 2015), only few studies have so far explored the neural mechanisms underlying age-related changes in this ability (Booth et al., 2003; Beaton et al., 2010; Wendelken et al., 2011; Vuontela et al., 2013; Hwang et al., 2014; Farrant and Uddin, 2015). These studies suggest that children as young as 8 years of age recruit fronto-parietal networks largely similar to adults during tasks requiring top-down control of attention. At the same time, however, both quantitative (Wendelken et al., 2011; Vuontela et al., 2013; Hwang et al., 2014) as well as qualitative (Konrad et al., 2005; Rubia et al., 2010) differences and differences with regard to functional connectivity (Hwang et al., 2014) in the recruitment of attention networks can be observed.

While empirical evidence regarding the neural basis of the development of selective attention is scarce, it is non-existent with regard to the development of attentional resolution. Also, research in adults has so far not produced a clear empirical basis regarding the neural mechanisms underlying the control of spatially precise attentional selection. Intriligator and Cavanagh (2001) suggested that the spatial extent of the selection region is controlled by the fronto-parietal attention network. Although this proposal has so far not been investigated directly, initial evidence for fronto-parietal involvement in spatiotemporal attentional precision comes from a study by Shim et al. (2009). This study demonstrates that increasing the speed of the items to be tracked (which – besides spatial attentional precision – is considered to be a parameter requiring higher attentional precision; Alvarez and Franconeri, 2007) results in increased activation in FEF and primary visual areas. Similarly, Drew et al. (2013) used event-related brain potentials to study the effect of increasing item speed during object tracking, and observed an increase in the contralateral delay activity – which supposedly originates from a source in the lateral intraparietal sulcus (Todd and Marois, 2004; Xu and Chun, 2006).

**FIGURE 1:**
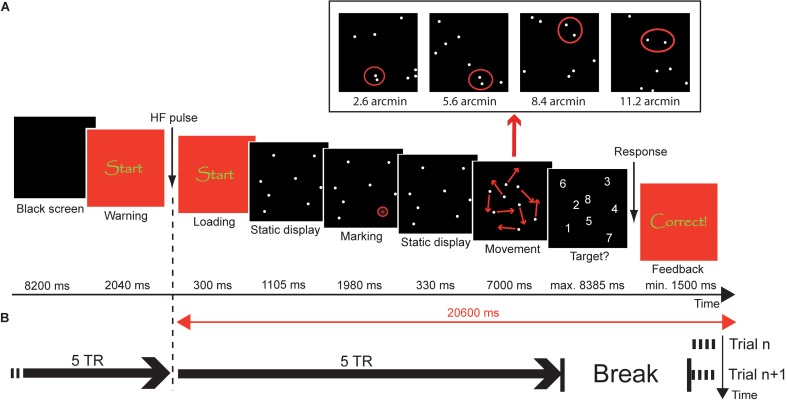
Object tracking task. **(A)** Trial procedure. At the beginning of the trial, a black screen was presented for 8.2 s, followed by a warning (2.34 s). Subsequently, a static display of 8 disks was presented for 1.105 s. Then, one of the disks was marked as target by highlighting it six times with a red circle (1.98 s). After another presentation of the static display for 330 ms, the disks moved in a pseudorandom manner for 7 s. The panel above the trial procedure illustrates the closest-possible encounters between target and a distractor during the tracking phase in the different distance conditions. After the tracking phase, the disks were replaced by digits (1–8) and the participant overtly named the disk that they thought was the target. The response was followed by a feedback display of variable duration, so that the time between loading of the tracking sequence and removal of the feedback was constant 20.6 s. **(B)** Timing of events. Each trial was a separate fMRI acquisition with a duration of 10 × TR = 25.6 s. Acquisition started during the feedback period of the preceding trial (compare Trial *n* vs. Trial *n*+1). Loading of the stimulus sequence was synchronized with the 6th TR.

In summary, there is relatively limited research on the neuro-cognitive development of visuo-spatial selective attention and a considerable lack of empirical work concerning the neural mechanisms underlying the resolution of the focus of attention and its development. The aim of the present study is to fill this gap by examining brain activation patterns during an object tracking task that systematically varies the required spatial precision of selective attention (**Figure 1**). To achieve this, the minimum distance between the to-be-tracked object and distractors was varied in four steps, i.e., 2.8, 5.6, 8.4, and 11.2 arcmin. Using functional magnetic resonance imaging (fMRI), BOLD activation changes were determined in children aged 7 years, 11 years, and in young adults, to identify brain mechanisms underlying the developmental changes in the resolution of spatial attention previously documented in behavioral work using the same paradigm (Wolf and Pfeiffer, 2014).

Based upon previous developmental studies on object tracking and voluntary attention (Booth et al., 2003; Beaton et al., 2010; Wendelken et al., 2011), it was hypothesized that all age groups would recruit the canonical network activated during object tracking (i.e., the fronto-parietal attention network – including the FEF and posterior parietal areas SPL and IPS – as well as motion area MT). At the same time, however, it was predicted that increasing age would be associated with activation increases within the fronto-parietal regions of this network during tracking. Increased demands with regard to spatial precision (i.e., reducing the minimum distance between the to-be-tracked object and distractors during the tracking phase) were expected to be associated with increased activity within the FEF and posterior parietal areas across all age groups (Shim et al., 2009; Drew et al., 2013). This increase in activity due to higher precision demands was expected to be the more pronounced the older the participants were.

## Materials and Methods

### Participants

Forty-nine healthy, right-handed participants from three age groups (7-year-olds, 11-year-olds, and adults) participated in the study. All participants were without history of neurologic/psychiatric disorders, developmental disorders of scholastic skills, or Attention Deficit Hyperactivity Disorder (ADHD). Letters were distributed to the parents of children in the first and fifth grades of several primary and secondary schools in Frankfurt (Germany) in order to recruit 7- and 11-year-olds. Participants of the adult sample were students of Goethe University Frankfurt. 11 children of the youngest group were removed from the sample because they either failed to complete the study (3 subjects) or because of excessive movement (8 subjects; see section on “fMRI Data Analysis” below for exclusion criteria). Two children of the older group were removed because of incomplete fMRI data. The final sample consisted of 35 participants: 12 7-year-old children (6 females; mean age: 7.4, range: 6.5–8.0), 11 11-year-old children (5 females; mean age: 11.5, range: 11.1–12.0), and 12 adults (6 females; mean age: 24.7, range: 21.4–34.1). This sample size was determined based on several considerations, including the moderate to strong effect sizes obtained in our previous behavioral study (Wolf and Pfeiffer, 2014), the fact that the object tracking paradigm generally elicits robust BOLD activation in attentional networks (e.g., Culham et al., 1998; Howe et al., 2009), but also the potential strain of the fMRI session for the youngest age group. Adults received monetary compensation or course credit for participation. Children received a gift token and a certificate of participation, and parents received monetary compensation for travel costs. The study was conducted in compliance with the Declaration of Helsinki and was approved by the Medical Ethics Committee of the University Hospital Frankfurt am Main. All participants or their parents gave written informed consent prior to testing.

Children and adults participated in a separate behavioral testing session before scanning. Right-handedness was assessed by the Edinburgh Handedness Inventory (Oldfield, 1971) and visual acuity of at least 20/20 was ensured by a visual acuity test with a standardized Landolt C eye chart (Schairer Ophthal-Technik, Stuttgart, Germany). Participants were screened for psychiatric conditions by using the Strengths and Difficulties Questionnaire (SDQ; Goodman, 1997) for the children samples and the Symptom Checklist-90-Revised (SCL-90-R; Franke, 2002) for the adult sample. Children were additionally rated for symptoms of ADHD by their parents on the ADHD subscale of a test for diagnosis of mental disorders in children and adolescents (DISYPS-KJ; Döpfner and Lehmkuhl, 2003). All participants had scores within ± 1 SD of the mean of a normative sample in the respective tests. IQ scores were estimated by using Raven’s Standard Progressive Matrices (SPM; Horn, 2009) for children and Standard Progressive Matrices-Plus (SPM-Plus; Raven et al., 2000) for adults. Mean IQ scores were 108 for 7-year-olds (*SD*: 11.05), 101 for 11-year-olds (*SD*: 14.22), and 108 for adults [*SD*: 11.65; *F*(2,37) = 1.67, *p* = 0.20].

### Experimental Procedures

On two occasions before the fMRI session, children were acquainted with the environment and sounds of an MRI scanner using a mock scanner set-up. During fMRI scanning, participants performed an object tracking task in which they had to track one target with focal attention in the presence of seven identically looking moving distractors for a time interval of 7 s (see **Figure 1**). Stimuli were presented with E-Prime 2.0 Professional (Psychology Software Tools, Inc., Sharpsburg, PA, United States) running on a PC (Shuttle Barebone SG31G2S, Intel Core 2 Duo, Windows XP) at a frame rate of 60 Hz. Images were projected onto a screen at the rear end of the MR scanner bore with an LCD projector (Sony, VPL-PX20) equipped with a custom-made lens. The screen could be seen by the participant on a mirror fastened to the head coil of the MR scanner. Each trial started with the presentation of a black screen (8.4 s) followed by a display indicating the immediate start of the task (2.34 s). Subsequently, a small quadratic black display (0.85°) containing eight static, near white disks (each 1.42 arcmin in diameter, RGB 200/200/200) was presented at the center of a dark gray background (RGB 30/30/30) for 1.105 s. During the next 1.980 s, one of the eight disks was marked as target by highlighting it six times for 165 ms with a red circle. After another 330 ms of the static display, the disks started to move in a pseudorandom manner.

During the movement sequences, the disks moved independently from each other. The rate of movement of each disk from one frame to the next (33 ms) was determined by a 2-component-vector *V* = (*x,y*) coding the horizontal and vertical movement of the disk. Both values of *V* ranged between -2 and +2 pixels, with positive values corresponding to rightward and upward movements and negative values corresponding to movements in the opposite direction. Each disk was assigned a random vector *V*_t_ = (*x,y*) at the start of the movement phase. This vector was conserved for at least five frames and then changed with a probability of 10% per frame. In case of a change of movement, both components of the vector were randomly and independently increased or decreased by the value ‘1’ [i.e., *V*_t+n_ = (*x*+/-1, *y*+/-1)]. If the value of one of the components exceeded ‘2,’ this component was reset to ‘2’ and if a disk was assigned *V* = (0,0), a new vector was calculated in order to prevent a halt of the disk. The disks bounced off the edges of the tracking field according to the law of reflection. In case of a collision between two disks, the disks repulsed each other at the specified distance and this repulsion also took place according to the law of reflection. Minimum center-to-center distance between two distractors (i.e., non-target stimuli) was a constant 4 arcmin throughout the whole experiment. In contrast, minimum center-to-center distance between the target and distractors was manipulated in four steps between trials (but kept constant within one trial). Specifically, target and distractor never came closer (center-to-center) than 2.8, 5.6, 8.4, or 11.2 arcmin, respectively.

Within each trial, movement of the target and the distractors had to meet a number of criteria apart from the minimum distances between targets and distractors. Those criteria were defined to ensure comparable movement of the disks in the different conditions. During one trial, there had to be exactly six target-distractor encounters and five collisions between the target and the edge of the tracking field. In addition, the tracking field was divided into six sections along both the horizontal and vertical axes, resulting in 36 quadrants. During each trial, the target had to visit exactly 16 of those quadrants (repeated visits possible). Movement sequences were generated ahead of the experiment using Java (Sun Microsystems, Santa Clara, CA, United States) and only sequences satisfying the criteria mentioned above were included in the experiment. The same movement sequences were presented to all participants.

After 7 s of movement, the disks stopped and were replaced by digits (1–8; 0.11°). Subjects were asked to verbally communicate the number that corresponded to the target’s position, during the silent period between two trials/fMRI runs (see below). The answer was transferred to the control room outside the scanner room by a MR-compatible microphone that was fastened to the headphones used for noise protection. Verbal responses were recorded digitally and monitored by the investigator who pressed the appropriate number on a keyboard connected to the PC that controlled stimulus presentation. The answers logged into the experimental software were later compared with the recorded response to determine the correctness of the participants’ responses. The investigator’s response terminated the presentation of the number display and a display containing visual feedback was presented. For the two older samples, the words “correct” and “incorrect” were displayed in German on the screen and the younger sample was additionally presented a green rectangle for a correct response and a red rectangle for an incorrect response. The maximum response interval was 8.385 s and the minimum time interval for feedback presentation was 1.500 s. In case of an earlier response, the duration of the feedback was prolonged so that total duration of the period between the loading of the tracking display and removal of the feedback display was 20.6 s. The feedback display was replaced by the black screen of the next trial.

Each trial was acquired as a separate functional MRI run to reduce head motion influences on fMRI data and to facilitate the acquisition of spoken responses without inducing additional motion artifacts. Data acquisition for each run started during the feedback phase of the previous run – to achieve steady state tissue magnetization before the start of the stimulus presentation of each trial – and stopped approximately 2 s after the presentation of the response display. Each functional run lasted for 25.6 s and was followed by a 5.44 s break during which participants gave their verbal response. Each trial thus had a fixed total duration of 31.04 s. Participants were explicitly instructed to withhold their response until the end of data acquisition of the current run to make their response audible. Temporal coordination between data acquisition and stimulus presentation during each trial/run was accomplished by time-locking the loading of the tracking display to the 6th TR. **Figure 1** illustrates the coordination of stimulus presentation and measurement.

Adults and 11-year-old children completed 60 experimental trials, i.e., 12 trials per distance condition (2.8, 5.6, 8.4, or 11.2 arcmin) and 12 additional trials of a baseline condition. However, the latter was not included into the present analysis of the experiment because it turned out not to be successful in suppressing tracking-related brain activations. 7-year-old children completed only three distance conditions, as a previous behavioral study had shown that 7-year-olds performed the task at chance level at 2.8 arcmin (Wolf and Pfeiffer, 2014). The trials for the two older samples were presented in four blocks of 15 trials (7 min 45 s each) and the trials of the youngest sample were presented in six blocks of eight trials (4 min 8 s each). Each block contained an equal number of trials of every distance condition. The sequence of trials within blocks was pseudorandomly determined such that the same condition was never repeated immediately, and fixed across subjects. The sequence of blocks was randomized across subjects. After each block, participants were offered to take a break of individual length. 7-year-old children were taken out of the scanner after half of the experiment to allow them to move. The entire measurement (excluding breaks and including the anatomical scan) lasted approximately 36 min for the two older samples and 30min for 7-year-old children.

To ensure children’s motivation, the task was embedded into a medieval scenario. According to this scenario, each target represented a thief that the participants had to track before they could finally convict him. For each successful conviction, participants were rewarded a virtual bag of gold coins. Before each break, they were reported their intermediate results and at the end of the experiment they were informed about their total result. All age groups underwent the same scenario. The introductory story was presented on a laptop computer before participants entered the scanner room, as well as five practice trials that could be repeated for 7-year-olds. Inside the scanner, subjects completed four further practice trials.

### fMRI Data Acquisition

During data acquisition, participants were instructed to lie as still as possible and their head movement was restricted by cushions. Earplugs and headphones dampened scanner noise. Functional images were acquired on a dedicated 3 Tesla head MRI scanner (Magnetom Allegra, Siemens, Erlangen, Germany) equipped with a Tx/Rx head coil, using a T2^∗^-weighted BOLD-sensitive gradient-echo echo planar imaging (EPI) sequence [repetition time (TR) = 2,560 ms, echo time (TE) = 30 ms] with 32 slices (thickness = 3 mm, gap = 1 mm, field of view = 192 mm × 192 mm, matrix size 64 × 64, in-plane resolution = 3 mm × 3mm, flip angle = 90°). Slices were tilted by 30° from axial toward coronal orientation to reduce susceptibility induced signal losses (Deichmann et al., 2003). The parameters governing the EPI readout were deliberately chosen to reduce acoustic scanner noise (band width 1,860 Hz/pixel, echo spacing 740 μs). To allow for exact timing, the fMRI sequence was modified in a way that after each acquisition of *N* consecutive volumes, a delay *T* was inserted, where *N* and *T* could be chosen freely by the researcher. For the parameters chosen in this study, each functional run (corresponding to one trial) consisted of *N* = 10 volumes (total acquisition time of 25.6 s; see above), followed by *T* = 5.44s (see above). The first two volumes of each run were discarded, restricting the analysis to fMRI data acquired under steady state conditions. After acquisition of the functional runs, a whole brain structural data set with 1 mm isotropic resolution was acquired, using a T1-weighted magnetization prepared-rapid gradient echo (MP-RAGE) sequence (TR = 1,700 ms, TE = 1.44 ms, TI = 800 ms, field of view = 256 mm × 256 mm × 160 mm, matrix size 256 × 256 × 160, flip angle = 7°).

### fMRI Data Analysis

Data were preprocessed and analyzed using Statistical Parametric Mapping (SPM; Wellcome Trust Center for Neuroimaging^1^). EPI images were motion corrected via realignment to the first image of the first run. To reduce residual errors after realignment, ArtRepair toolbox for high motion samples (Stanford Psychiatric Neuroimaging Laboratory^2^) was applied to the EPI data sets that had been prepared by a first step of spatial smoothing (4 mm FWHM). This algorithm removes interpolation errors from the image time series on a voxel-by-voxel basis (Grootoonk et al., 2000) and has been recommended in preference to adding motion regressors to the design matrix in high motion samples (Mazaika, 2009; Wilke, 2012; Fassbender et al., 2017). In a second step, ArtRepair was used to identify noisy volumes on the basis of frame-to-frame movement (threshold: 1.5 mm, calculated using Pythagoras’ theorem based on the 6 translation and rotation parameters derived from SPM’s image realignment process; Mazaika, 2009; Wilke, 2012, 2014; Power et al., 2015) and signal fluctuations, which were then corrected via interpolation between the nearest non-repaired scans (“scrubbing”; Power et al., 2012, 2015; Siegel et al., 2014). Trials containing more than two interpolated volumes and participants with more than 15% corrected trials across the whole experiment were excluded from further analysis (see Turner and Miller, 2013; Fassbender et al., 2017 for comparable thresholds). Subsequently, the functional data were normalized (MNI152) and further smoothed (7 mm FWHM), resulting in a final spatial smoothness of 8 mm FWHM.

At the first level of statistical analysis, a general linear model (GLM) was calculated in which each run (i.e., trial) was modeled by a separate regressor with a square wave representing the tracking period (high-pass filter 128 s cut-off). Contrast estimates were computed for each subject by contrasting parameter estimates (beta images) of all runs belonging to one condition with parameter estimates of all runs of another condition or with zero, respectively. Resulting contrast images were submitted to random-effects group analyses by performing *t*-tests (to determine the significance of a certain contrast across all participants) or full-factorial ANOVA (to compare age groups). Activations are reported at a family-wise corrected significance level of 0.05, by combining a voxel-level *p*-value of 0.001 (uncorrected) with a cluster-extent threshold (*k* = 62) determined by a Monte-Carlo simulation (10,000 iterations) using the AFNI routine 3dClustSim^3^ (voxel size 2 mm × 2 mm × 2 mm; 2nd nearest neighbor clustering). For a detailed characterization of the patterns of developmental changes in significant brain regions, spheres (diameter: 12 mm) were centered on peak voxels of clusters showing age effects; subsequently, mean beta values across all voxels included in the sphere were extracted and used to visualize developmental trends and to conduct direct mean comparisons between the three age groups.

## Results

### Behavioral Results

**FIGURE 2:**
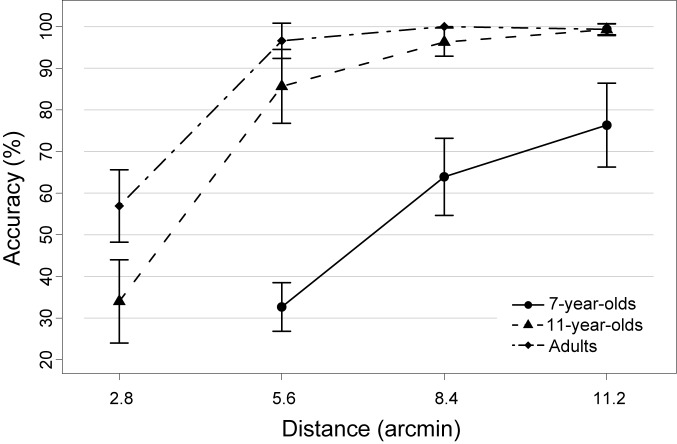
Mean accuracy of each age group as a function of target-distractor-distance in the object tracking task. Note that the most difficult condition (i.e., 2.8 arcmin) was not acquired in the group of 7-year-old children, as it was too difficult for children of that age. Error bars represent 95% confidence intervals.

Accuracy (i.e., the probability of picking the correct item after the 7 s tracking period; chance level = 12.5%) increased with increasing age and increasing target-distractor-distance (**Figure 2**). Two separate repeated measures ANOVAs were calculated to statistically support this observation, which was necessary because the most difficult condition (2.8 arcmin) was not acquired in the youngest participant group due to its difficulty (see section “Materials and Methods”). The first analysis compared all three age groups in those conditions completed by all groups, i.e., 5.6, 8.4, and 11.2 arcmin. Accuracy increased with age [*F*(2,32) = 95.85, *p* < 0.0001], with significant increases between all age groups [both *t*(21) > 2.6, *p* < 0.025; Bonferroni corrected significance threshold 0.05/2 = 0.025]. Reduced performance for 7-year-olds compared to 11-year-olds and adults was visible even in the easiest condition (11.2 arcmin; both *t* > 4, *p* < 0.0001). In addition, there was a significant main effect of distance [*F*(2,64) = 49.44, *p* < 0.0001] reflecting that accuracy increased with distance [both *t*(34) > 2.5, *p* < 0.025]. A robust age group × distance interaction [*F*(4,64) = 18.21, *p* < 0.0001] reflects that the distance effect is driven by the 7-year-olds [5.6 vs. 8.4 arcmin: *t*(11) = 8.75, *p* < 0.0001; 8.4 vs. 11.2 arcmin: *t*(11) = 2.57; *p* = 0.026] while 11-year-olds and adults showed no distance-dependent improvements between these distance conditions (all *t* < 2.52).

In a second ANOVA, performance across all four distance conditions was compared, but only for the two older samples. Again, better performance was observed in adults than in 11-year-olds [*F*(1,21) = 12.91, *p* < 0.005] as well as a performance increase with increasing distance [*F*(3,63) = 173.18, *p* < 0.0001]. Increases in accuracy were observed between 2.8 and 5.6 arcmin distance [*t*(22) = 13.37, *p* < 0.0001] and between 5.6 and 8.4 arcmin [*t*(22) = 2.89, *p* < 0.01], but not between 8.4 and 11.2 arcmin [*t*(22) = 1.79, *p* = 0.09; see also previous paragraph]. Optimal performance was thus reached by both older groups already at a distance of 8.4 arcmin. An age group x distance interaction [*F*(3,63) = 6.66, *p* < 0.01] was due to the fact that 11-year-olds and adults only differed in performance in the closest distance condition [2.8 arcmin; *t*(21) = 3.40, *p* < 0.0125; all other *p* > 0.0125; Bonferroni corrected significance threshold 0.05/4 = 0.0125].

### Age-Independent Tracking-Related Brain Activations

**FIGURE 3:**
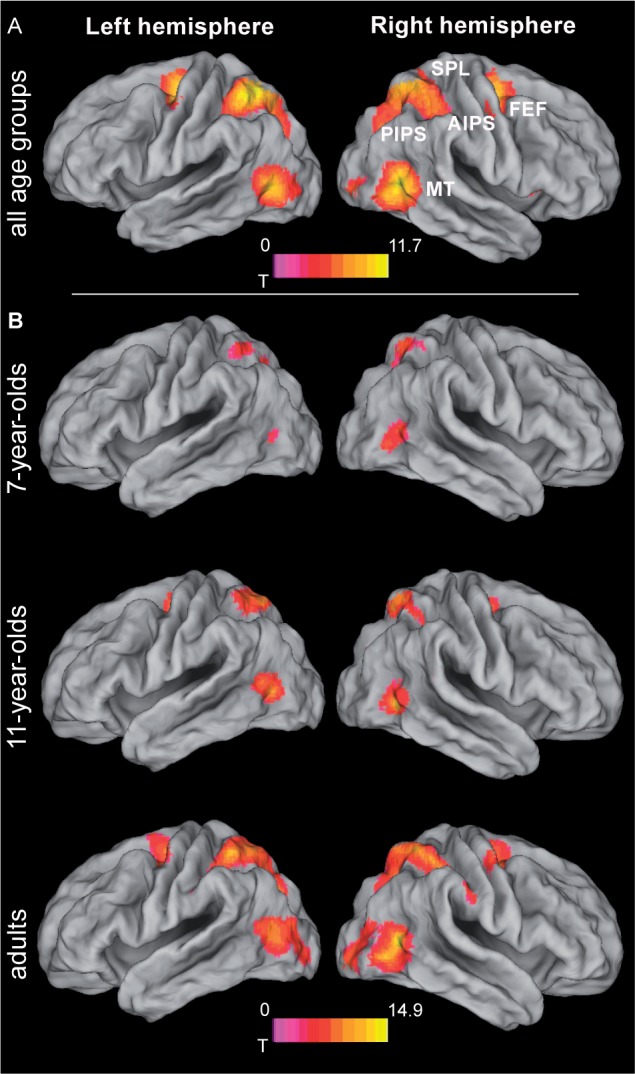
Brain activation during object tracking. **(A)** Simple effect of tracking across all age groups, i.e., largest distance condition (11.2 arcmin) tested against zero. See **Table 1** for further details. **(B)** Age group specific brain activations during tracking. Statistical threshold: *p* < 0.05 (FEW corrected; cf. section “Materials and Methods”). AIPS, anterior intraparietal sulcus; FEF, frontal eye fields; MT, motion area MT/V5; PIPS, posterior intraparietal sulcus; SPL, superior parietal lobe.

To determine regions showing age-independent activation during the tracking of moving objects, we assessed activation in the largest-distance tracking condition (11.2 arcmin) relative to zero and independent of age group. In developmental neurocognitive research, differences in activation between age groups are often accompanied by differences in task performance, which leaves open the possibility of attributing differences in neural activation to either group membership or level of performance or both (Church et al., 2010). Accounting for performance differences is thus an important prerequisite for isolating the unique effects of age, which was accomplished here by including performance as a covariate of no interest. Across age groups, tracking activated an extended bilateral fronto-parietal network (**Figure 3A** and **Table 1**; for age group-specific brain activations, see **Figure 3B**) consisting of the SPL, aIPS and pIPS in parietal cortex, FEF, and MT. In addition, right anterior insula and subcortical areas including putamen and claustrum, and the cerebellum were activated during tracking of moving objects.

**Table 1:** Simple effect of object tracking (greatest distance condition (11.8 arcmin) > zero) across all age groups.

					*MNI coordinates*		
					
*Anatomical region*	*Hem*	*BA*	*k*	*T*_max_	*x*	*y*	*z*
Anterior insula, inferior frontal gyrus, putamen, claustrum	R	13	314	7.05	38	16	8
Precentral gyrus, superior frontal gyrus, medial superior frontal gyrus, middle frontal gyrus	R	6/24/32	1347	10.47	24	-10	50
Precentral gyrus, superior frontal gyrus, medial superior frontal gyrus, supplementary motor area, middle frontal gyrus	L	24	1152	10.66	-28	-12	48
Precentral gyrus, inferior frontal gyrus	R	6/9	127	5.00	52	2	32
Superior parietal lobe, anterior intraparietal sulcus, inferior parietal lobe, postcentral gyrus, precuneus, posterior intraparietal sulcus, middle temporal gyrus, middle occipital gyrus	L	5/7/19/37/39/40	3447	13.80	-20	-56	56
Middle temporal gyrus, inferior temporal gyrus, middle occipital gyrus, posterior intraparietal sulcus, precuneus, inferior parietal lobe, superior parietal lobe, anterior intraparietal sulcus, paracentral lobe, postcentral gyrus	R	5/7/19/37/39/40	4716	11.82	46	-60	6
Cerebellum (not depicted in **Figure 3A**)	L		106	5.78	-24	-40	-42
	L/R		75	4.11	-2	-74	-32


**BA, approximate Brodmann’s area; Hem, hemisphere; L, left; R, right; MNI, coordinates referring to the Montreal Neurological Institute template brain included in the SPM software package; T_*max*_, maximum t statistic in the cluster; k, cluster size in voxels. Activation is reported for a threshold of p < 0.05 (corrected), constituted by a voxel-level threshold of p < 0.001 and a cluster-level threshold of k > 62 (determined using 3dClustSim)*.*

### Age Effects on Tracking-Related Brain Activations

**FIGURE 4:**
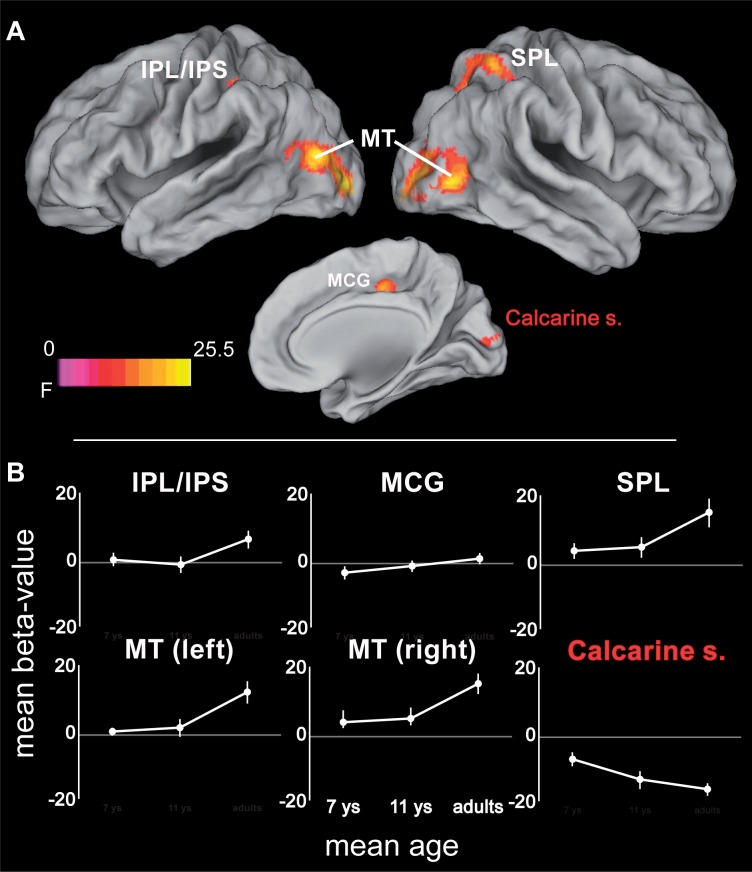
**(A)** ANOVA (*F*-statistic) results for age differences in tracking-related brain activation: middle cingulate gyrus (14, -18, 44; *F*_max_ = 16.6), left inferior parietal lobe/intraparietal sulcus (-42, -38, 46; *F*_max_ = 14.14), right superior and inferior parietal lobe (34, -50, 58; *F*_max_ = 17.5), middle and inferior occipito-temporal cortex (right: 46, -70, 0; *F*_max_ = 23.34; left: -44, -78, 12; *F*_max_ = 25.51), calcarine sulcus (10, -90, 12; *F*_max_ = 17.01). **(B)** Mean beta-values for the different age groups for the regions showing age-related differences in tracking. Error bars represent the 95% confidence intervals. Statistical threshold: *p* < 0.05 (FEW corrected; cf. section “Materials and Methods”). AIPS, anterior intraparietal sulcus; FEF, frontal eye fields; IPL/IPS, inferior parietal lobe and intraparietal sulcus; MCG, mid-portion of cingulate gyrus; MT, motion area MT/V5; PIPS, posterior intraparietal sulcus; SPL, superior parietal lobe. White labels indicate activation increases; red label indicates activation decrease. See **Table 2** for further details.

While all age groups thus recruit a similar network during tracking, we also expected age-related changes within this network. Age-related differences (again controlling for performance differences) were observed in bilateral MT extending into occipital areas, right SPL extending into IPS and precuneus, left inferior parietal lobule (IPL)/IPS, the mid-portion of the right cingulate gyrus, and in right primary visual cortex (**Figure 4** and **Table 2**). Parietal areas and visual area MT showed a common developmental increase of activation magnitude between 11 years of age and adulthood [all *t*(21) > 4, *p* < 0.0001], whereas there were no activation differences between 7 and 11 year of age [all *t*(21) < 1, *p* > 0.34]. Developmental effects deviated from this pattern in the mid-cingulate gyrus, which showed a significant monotonic age-related increase between 7-year-olds and adults [7 years vs. adults: *t*(22) = 3.80, *p* < 0.005; 7 years vs. 11 years: *t*(21) = 1.69, *p* = 0.11; 11 years vs. adults: *t*(21) = 2.33, *p* = 0.03; Bonferroni corrected significance threshold 0.05/3 = 0.017]. Activation in primary visual cortex around the calcarine sulcus showed the reverse pattern and decreased between 7 and 11 years of age [*t*(21) = 4.04, *p* < 0.005] and, at trend level, between age 11 and adults [*t*(21) = 2.05, *p* = 0.053].

**Table 2:** Age differences in activation elicited by object tracking.

					*MNI coordinates*		
					
*Anatomical region*	*Hem*	*BA*	*k*	*F*_max_	*x*	*y*	*z*
Middle cingulate gyrus	R	24/31	175	16.60	14	-18	44
Inferior parietal lobe, intraparietal sulcus	L	40	113	14.14	-42	-38	46
Superior parietal lobe, anterior intraparietal sulcus, inferior parietal lobule, precuneus, superior occipital gyrus	R	7/39/40	570	17.50	34	-50	58
Middle temporal gyrus, inferior temporal gyrus, middle occipital gyrus, superior occipital gyrus, inferior occipital gyrus, lingual gyrus	R	18/19/37/39	1296	23.34	46	-70	0
Middle temporal gyrus, middle occipital gyrus, inferior occipital gyrus, lingual gyrus	L	18/19/39	725	25.51	-44	-78	12
Calcarine sulcus	R	17/18	78	17.01	10	-90	12


**BA, approximate Brodmann’s area; Hem, hemisphere; L, left; R, right; MNI, coordinates referring to the Montreal Neurological Institute template brain included in the SPM software package; F_*max*_, maximum F statistic in the cluster; k, cluster size in voxels. Activation is reported for a threshold of p < 0.05 (corrected), constituted by a voxel-level threshold of p < 0.001 and a cluster-level threshold of k > 62 (determined using 3dClustSim)*.*

### Age-Independent Mechanisms Underlying the Resolution of Attention

Investigating the neural mechanisms underlying increasing spatial precision of tracking, i.e., the resolution of the focus of attention, required contrasting two conditions with differences in performance that are as large as possible (thus assuring an actual difference in task difficulty) while avoiding floor effects in performance to assure considerable effort even in the easier distance condition. Critically, the difference in performance of the different age groups needed to be comparable. These criteria were met for different pairs of conditions for the different age groups, i.e., 2.8 > 5.6 arcmin for adults and 11-year-olds, and 5.6 > 11.2 arcmin for 7-year-old children. A 2 (distance: large vs. small) × 3 (age group) ANOVA showed that performance differences (7-year-olds 43.75%, 11-year-olds 51.52%, adults 39.58%) between these distance conditions did not differ statistically between age groups [*F*(2,32) = 1.50, *p* = 0.24]. Absolute performance levels (i.e., average of easy and difficult conditions), however, differed between age groups [*F*(2,32) = 12.12, *p* < 0.001]. This difference was accounted for by including the average performance level as a covariate of no interest into fMRI analyses.

**FIGURE 5:**
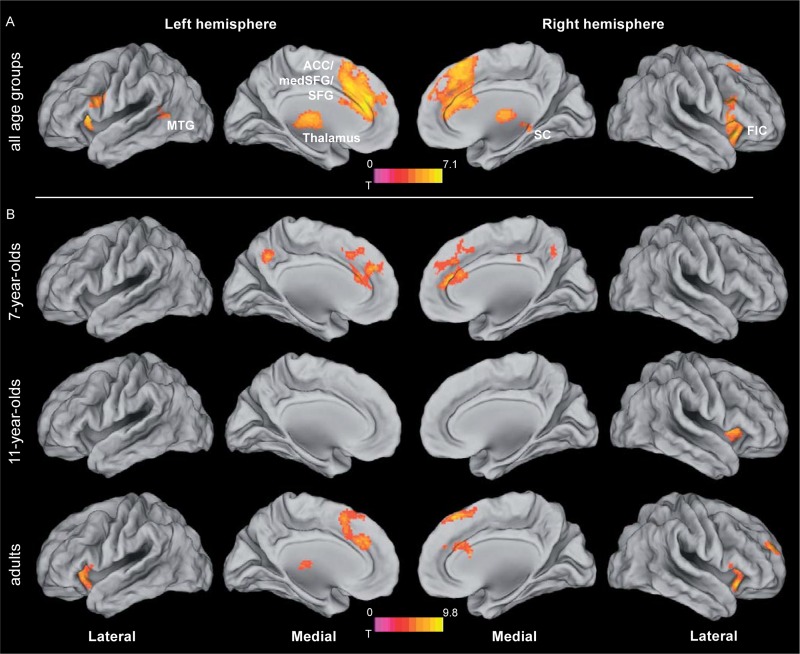
Brain activation related to attentional precision. **(A)** Activation related to increasing the spatial precision of the focus of attention, independent of age group. For the different age groups, contrasts of different difficulty conditions (i.e., 2.8 > 5.6 arcmin for adults and 11-year-olds, and 5.6 > 11.2 arcmin for 7-year-old children) were fed into the analysis. For the rationale behind the choice of the respective difficulty conditions, see section “Age-Independent Mechanisms Underlying the Resolution of Attention”. See **Table 3** for further details. **(B)** Age group specific brain activations related to increasing spatial precision (based on the same contrasts of different difficulty conditions for the different age groups as depicted in *A*). Statistical threshold: *p* < 0.05 (FEW corrected; cf. section “Materials and Methods”). ACC, anterior cingulate cortex; FIC, fronto-insular cortex; MTG, middle temporal gyrus; SC, superior colliculus; SFG, superior frontal gyrus.

Increasing the demands on the spatial precision of selective attention yielded increased activation bilaterally fronto-insular cortex (FIC) and basal ganglia. In addition, a large bilateral cluster containing anterior cingulate cortex (ACC), medial and lateral superior frontal gyrus and supplementary motor area, as well as a cluster containing thalamus and the superior colliculi were increasingly activated with increasing demands on spatial precision (**Figure 5A** and **Table 3**; for age group-specific brain activations, see **Figure 5B**).

**Table 3:** Activation related to increased demands in precision (i.e., 2.8 > 5.6 arcmin for adults and 11-year-olds, and 5.6 > 11.2 arcmin for 7-year-olds) across all age groups.

					*MNI coordinates*		
					
*Anatomical region*	*Hem*	*BA*	*T*_max_	*k*	*x*	*y*	*z*
Anterior insula, claustrum, putamen, inferior frontal gyrus, precentral gyrus	L	6/9/13/44/45/47	7.09	901	-34	22	8
Anterior insula, claustrum, putamen, inferior frontal gyrus, precentral gyrus, middle frontal gyrus	R	9/13/44/45/46/47	6.69	1008	30	20	-6
Anterior cingulate cortex, medial superior frontal gyrus, superior frontal gyrus, supplementary motor area, middle cingulate cortex	R/L	6/8/9/10/24/32/33	6.98	4347	-8	38	26
Middle temporal gyrus	R	21	4.17	82	56	-30	-8
Middle temporal gyrus, superior temporal gyrus	L	22/39	4.05	108	-54	-56	10
Thalamus, superior colliculi, midbrain	L/R		5.41	1294	-8	-14	8
Striatum	R		3.89	70	14	12	4
Cerebellum	L		4.17	128	-18	-50	-34


**BA, approximate Brodmann’s area; Hem, hemisphere; L, left; R, right; MNI, coordinates referring to the Montreal Neurological Institute template brain included in the SPM software package; T_*max*_, maximum t statistic in the cluster; k, cluster size in voxels. Activation is reported for a threshold of p < 0.05 (corrected), constituted by a voxel-level threshold of p < 0.001 and a cluster-level threshold of k > 62 (determined using 3dClustSim)*.*

### Age Effects on Brain Systems Underlying Attentional Resolution

**FIGURE 6:**
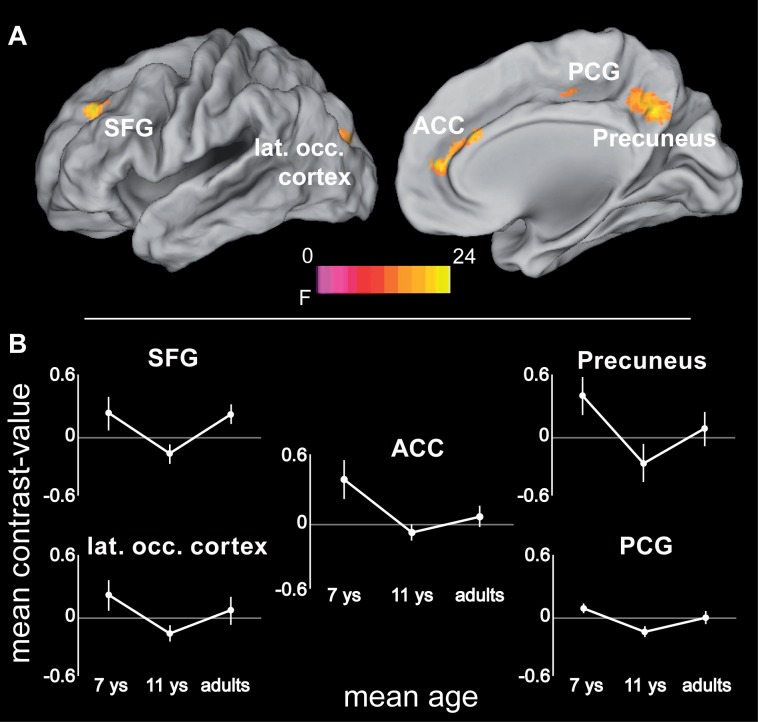
**(A)** ANOVA (*F*-statistic) results of age differences for increased demands in attentional precision (again based on the contrasts 2.8 > 5.6 arcmin for adults and 11-year-olds, and 5.6 > 11.2 arcmin for 7-year-old children): Left superior and middle frontal gyrus (-24, 40, 36; *F*_max_ = 15.86), anterior cingulate cortex (14, 50, 12; *F*_max_ = 19.5), left posterior cingulate gyrus (-26, -36, 36; *F*_max_ = 23.97), precuneus (4, -56, 36; *F*_max_ = 15.4), left superior/middle occipital gyrus (-20, -88, 22; *F*_max_ = 11.69). **(B)** Mean contrast-values (i.e., difference in beta-values between the respective easy and difficult tracking condition) for the regions showing age-related differences with increasing spatial precision. Error bars represent the 95% confidence intervals. Statistical threshold: *p* < 0.05 (FEW corrected; cf. section “Materials and Methods”). ACC, anterior cingulate cortex; PCG, posterior cingulate gyrus; SFG, superior frontal gyrus. See **Table 4** for further details.

In a final step, age differences in the neural substrates underlying increased spatial precision were examined, again taking into account differences in absolute performance levels. One area was found within the described network for increased spatial precision, this was the ACC. Here, precision-related brain activation dropped between 7 and 11 years of age [*t*(21) = 4.92, *p* < 0.0001], while ACC activity in 11-year-olds and adults differed only at trend level [*t*(21) = 2.34; *p* = 0.029, Bonferroni corrected threshold 0.05/2 = 0.025; **Figure 6** and **Table 4**]. In addition, four areas that belonged neither to the precision-related network nor to the fronto-parietal tracking network (i.e., superior frontal gyrus, posterior cingulate gyrus, precuneus, and postero-lateral occipital cortex) showed a comparable activation decrease between 7 and 11 years of age [all *t*(21) > 4.14, *p* < 0.0001], but an increase between 11 years of age and adulthood [all *t*(21) > 2.97; *p* < 0.009].

**Table 4:** Age differences in activation elicited by increased demands in precision (based on contrasts for difficulty conditions 2.8 > 5.6 arcmin for adults and 11-year-olds, and 5.6 > 11.2 arcmin for 7-year-olds; cf. section “Age-Independent Mechanisms Underlying the Resolution of Attention”).

					*MNI coordinates*		
					
*Anatomical region*	*Hem*	*BA*	*k*	*F*_max_	*x*	*y*	*z*
Anterior cingulate cortex, medial superior frontal gyrus	R	10/32	193	19.50	14	50	12
Superior frontal gyrus, middle frontal gyrus	L	9	64	15.86	-24	40	36
Posterior cingulate gyrus, postcentral gyrus, precentral gyrus, middle frontal gyrus	L	31	469	23.97	-26	-36	36
Precuneus, posterior cingulate gyrus, middle cingulate gyrus	R/L	7/31	535	15.40	4	-56	36
Superior occipital gyrus, middle occipital gyrus, middle temporal gyrus	L	18/19	122	11.69	-20	-88	22


**BA, approximate Brodmann’s area; Hem, hemisphere; L, left; R, right; MNI, coordinates referring to the Montreal Neurological Institute template brain included in the SPM software package; F_*max*_, maximum F statistic in the cluster; k, cluster size in voxels. Activation is reported for a threshold of p < 0.05 (corrected), constituted by a voxel-level threshold of p < 0.001 and a cluster-level threshold of k > 62 (determined using 3dClustSim)*.*

## Discussion

In this study, we investigated both the neural mechanisms underlying the resolution of attention and their developmental change from childhood to adulthood. For this purpose, we examined brain activation (i.e., BOLD signals) during a visual object-tracking task that requires increasingly fine-grained spatial precision cross-sectionally in children aged 7 years, children aged 11 years, and young adults. Across all age groups, performance dropped with increasing proximity between target and distractors, and thus with the requirement of increasingly narrowing down the focus of attention. Adults and 11-year-old children could sustain high performance up to a higher level of required precision. Given the similar performance patterns across difficulty levels of 11-year-olds and adults, the behavioral data of this study indicate that the major development in the ability to constrict the attentional focus is achieved between 7 and 11 years of age (which is consistent with our previous behavioral work; Wolf and Pfeiffer, 2014).

Object tracking activated a canonical tracking network (Culham et al., 1998; Jovicich et al., 2001; Howe et al., 2009; Alnaes et al., 2015) that largely coincides with established attention systems (Moore and Zirnsak, 2017), already for 7-year-old children. A subset of these areas – i.e., right SPL, left IPL/IPS, and bilateral area MT – were recruited during tracking to a higher degree with increasing age, while a region around the calcarine sulcus showed a developmental decrease in activation. Increasing the resolution of spatial attention – i.e., narrowing the focus of attention – involved a distinct set of brain areas, particularly the FIC and ACC, the superior colliculi, thalamus, and striatum. Developmental changes in the neural mechanisms underlying an increased resolution of the attentional focus were seen in only one of these areas, i.e., in the ACC, where an age-related reduction was observed. In the following, we discuss several implications of these results for understanding the development of visuo-spatial attention and of the ability to control the size of the focus of attention.

### Neurocognitive Development of Object Tracking Networks

Within the attentional tracking network, parietal regions and motion area MT feature a protracted development that showed the most pronounced age-related increase in tracking-related activation between 11 years of age and adulthood. It can be excluded that this developmental pattern reflects performance changes rather than maturation of neural systems, as (i) the most pronounced performance improvements occurred between 7 and 11 years of age, which is distinct from the developmental pattern in brain activations, and (ii) performance differences were statistically controlled for in the fMRI analyses. The protracted neurocognitive development of attention-related temporal and parietal systems clearly dissociates from the developmental pattern seen in primary visual cortex, which shows age-related decrease of tracking-related activity, particularly between 7 and 11 years of age.

Based on this dissociation of developmental changes in primary visual vs. attention-related brain systems, three conclusions can be derived. First, our findings confirm and extend earlier work showing activation of the canonical fronto-parietal attention network in 11-year-old children during tracking (Beaton et al., 2010) and generally a large degree of accordance between children’s and adults’ activations during the deployment of voluntary, i.e., goal-driven, attention (Booth et al., 2003; Wendelken et al., 2011; Vuontela et al., 2013). Second, the observation of increasing involvement of PPC with age (see also Olesen et al., 2006; Velanova et al., 2008; Wendelken et al., 2011) suggests an increasing reliance on parietal sites and the functions they provide, as the attention system matures. Jointly with the FEF, the PPC is assumed to form a priority map of the visual field (Baluch and Itti, 2011), i.e., a topologically organized map of the external environment encoding the relative importance of objects or events occurring at the corresponding location of the visual scene (Gottlieb et al., 1998; Thompson and Bichot, 2005; Gottlieb and Balan, 2010). Activity in this map is supposed to be driven both by salient (i.e., capturing attention in a bottom-up manner) external stimuli (Santangelo, 2015) and by the demands of the task at hand, and is assumed to guide attention or other goal-driven behavior toward the locations/objects of high relevance (Gottlieb and Balan, 2010; Nardo et al., 2011). Within the fronto-parietal priority network, the unique function of PPC seems to be to bind visuo-spatial, motor, and cognitive information to eventually deliver a spatial priority representation that can be easily translated into action (Gottlieb, 2007). As they become older, children may make increasing use of this complex representation to optimize their performance in attention-demanding tasks.

Third, our data indicate a qualitative developmental change among visual systems recruited to support attentionally guided visual processing from a reliance on primary visual areas by the youngest age group toward more task-optimized processing in higher-order visual area MT by the older age groups. The developmental decrease in activation of primary visual areas – that mature earlier during development (Conner et al., 2004) – suggests that the children (from both age groups) relied more on lower-level visual representations coded in this region to accomplish the task at hand, whereas adults seemed to rely more on the higher-level, motion-sensitive area MT where we observed an age-dependent increase in activation. Lower activation of MT by younger participants suggests that it may not be fully matured at the age of our samples of children – which has indeed been demonstrated both functionally (Klaver et al., 2008) and structurally (Sowell et al., 2003). The findings of this study thus extend the proposal of Klaver et al. (2008), who suggested that with development, higher-level visual processes increasingly substitute lower-order visual-perceptual processes, to the domain of attention.

The neuronal changes observed during tracking of objects may also partly be accounted for by age-dependent differences in spatial processing abilities (Jahn et al., 2012; Brockhoff et al., 2016). It has been shown that these abilities develop markedly during the age range examined here (Stiles et al., 2013). More specifically, younger children (4–6 years of age) represent objects within topological (i.e., non-metric) space and bind single objects to places, whereas older children (8–10 years of age) represent objects within Euclidean (i.e., metric) space and group objects into areas determined by vectors of a spatial axis system (Lange-Küttner, 2006; Lange-Küttner and Küttner, 2015). It can be assumed that children in our study switched from a predominantly topological, one object-in-place representation of space at 7 years of age to a metric representation where objects are grouped into areas at 11 years of age. If this were the case, the age-related changes observed in neuronal activation patterns may (at least partly) be related to this transition. While there is, to our knowledge, yet no developmental evidence regarding the neuronal correlates of different spatial representations, there is some neurocognitive research on the development of a spatial ability that is conceptually closely related to the idea of processing objects as object-in-place units or as objects grouped into areas, i.e., the concept of local vs. holistic processing (Navon, 1977). This research proposes that in hierarchical configurations of stimuli, adults have a strong (attentional) bias to process configurations on a global level (i.e., the whole configuration), whereas children younger than 10 years predominantly process these configurations on a local level (i.e., on a single-unit level; Dukette and Stiles, 2001; Harrison and Stiles, 2009). Interestingly, the shift from (predominantly) local to holistic processing seems to be related to morphometric gray matter changes in regions similar to the ones identified in the present study, i.e., the fronto-parietal attention network and primary visual areas (Poirel et al., 2011, 2014). This observation leaves open the possibility that some of the changes observed in the present study, both behaviorally and at the level of brain activation, may be interpreted in terms of changes in the computations underlying different types of spatial processing. Under this perspective, younger participants who display a bias toward local processing might predominantly process the objects in the display in a single-unit manner – which might be mediated by stronger involvement of primary visual areas. Older participants, in contrast, might rather process the whole spatial configuration relying on an increasingly mature fronto-parietal network. Further research is yet required to investigate this idea.

### Neural Mechanisms Underlying the Resolution of the Focus of Attention

While tracking-related activity was mainly seen in the fronto-parietal attention network, the set of brain regions that were evoked to achieve increased spatial precision (i.e., FIC, medial and lateral superior frontal cortex, and thalamus and superior colliculi) largely conforms to a different functional network that has been identified in the investigation of resting-state fMRI connectivity, i.e., the so-called *salience network* (Seeley et al., 2007). This network is thought to highlight the (neural) salience of external stimuli by increasing the amount of cognitive resources available for their processing (Menon and Uddin, 2010; Uddin, 2015). This is supposedly achieved by control signals from the salience network to the fronto-parietal attention network, which in turn effects the stimulus highlighted by the salience network to have preferential access to the brain’s attentional and memory resources (Sridharan et al., 2008; Menon and Uddin, 2010; Chen et al., 2013, 2016; Goulden et al., 2014). Given that increased attentional precision in the present study lead to increased activity in the upstream salience network independent of age group, an important conclusion from our data may thus be that willingly increasing the spatial precision of attention is a function of the insula-cingulate salience network rather than the fronto-parietal attention network.

Within this network, the different components are ascribed distinct and finely coordinated functions (Menon and Uddin, 2010). The FIC is assumed to be responsible for the detection of behaviorally relevant or perceptually salient stimuli (Seeley et al., 2007; Uddin, 2015), that is stimuli that are either of high relevance for current behavioral goals or that exhibit a high degree of sensory prominence relative to their background (Santangelo, 2015). The FIC is supposed to be the ultimate source of the control signals (Crottaz-Herbette and Menon, 2006; Chand and Dhamala, 2016b) which are then passed on to ACC (Menon and Uddin, 2010). The ACC is thought to sustain the control signals initiated by the FIC and to promote those signals firstly to the components of the fronto-parietal network and secondly to cortical areas related to motion and action (Crottaz-Herbette and Menon, 2006; Menon and Uddin, 2010; Chand and Dhamala, 2016a). The fact that in the data presented here activation around ACC spreads into motor areas like SMA appears compatible with this assumption. Besides FIC and ACC, the superior colliculi – that are not considered part of the salience network – also showed increased activity during increased attentional precision. Despite their subcortical location, the superior colliculi are in fact generally considered to be an integral part of the fronto-parietal attention network (Lovejoy and Krauzlis, 2010; Baluch and Itti, 2011). Recent evidence further suggests that the superior colliculi work in tight coupling with the thalamus – a supposed subcortical component of the salience network (Seeley et al., 2007) – to convey attentional control (Wurtz et al., 2011). We thus suggest that the modulation of visual processing required during increased spatial precision is not accomplished by the cortical components of the dorsal fronto-parietal attention network but by a routing of control signals generated by the salience network via the thalamus and superior colliculi.

### Neurocognitive Development of the Resolution of the Focus of Attention

The networks recruited to handle increasing attentional precision demands are remarkably similar in children and adults: already participants from the 7-year-old sample activated the salience regions discussed above as well as the superior colliculi and thalamus. This result is consistent with developmental functional connectivity studies suggesting that the basic organization of the salience network is similar to adults by the age of seven (Fair et al., 2012b; Power et al., 2012; Supekar and Menon, 2012; Grayson and Fair, 2017).

The results of this study deviate from earlier studies that observed either a decrease (Supekar and Menon, 2012) or an increase in FIC function with age in tasks requiring cognitive control (for a review see Houdé et al., 2010). Instead an astonishing stability of FIC activation across age was observed, which suggests mature FIC function already from an early age. In contrast, under increased spatial precision demands, increased activation in the most anterior part of ACC was observed in the 7-year-old sample compared to the two older samples.

Findings from research on the development of functional networks may help to elucidate this developmental change. Several studies have demonstrated that while the salience network’s basic structure in children is similar to that of adults, refinements take place as to the salience network’s within- and between-network connectivity in several regards (Fair et al., 2012a,b; Gu et al., 2015; Marek et al., 2015). Firstly, studies have demonstrated that brain development between childhood and adulthood is characterized by a trend from local interconnectivity in children to long-range functional coupling in adults (Fair et al., 2009). More specifically, these studies show that connectivity between anatomically close regions of interest slowly decreases until adulthood whereas long-range connectivity, especially anterior-posterior links, gradually increases. Secondly, the interplay between FIC and ACC (with the FIC being a major source of control signals for ACC in adults; Menon and Uddin, 2010) seems to change between childhood and adulthood in that the directed causal influence of FIC on ACC has been found to increase between childhood and adulthood (Supekar and Menon, 2012). Thirdly, the salience network as a whole as well as its individual components show age-related changes in integration with other networks, especially the somatomotor network presumably resulting in a more rapid access from the salience network to the motor system to control goal-directed behavior (Grayson et al., 2014; Marek et al., 2015). In sum, these results indicate that a ‘calibration’ of ACC function both with regard to within-network as well as with regard to between-network interaction takes place during childhood (and into adulthood). We speculate that these refinements come along with altered ACC activation in younger children. The different empirical findings offer different possible explanations. Increased ACC activation in this age group may be a result of stronger local frontal connectivity entailing stronger frontal activations to accomplish the computations that adults conduct by relying more on long-distance networks. Alternatively it may be a consequence of weak control signals from FIC that necessitate stronger involvement of the ACC to compensate for weaker input from upstream FIC or it may be related to stronger effort to forward information to downstream networks like the somatomotor network. The appropriateness of these proposals will have to be determined by further research.

Relating the changes in ACC activation to behavioral development, the aforementioned spatial processing framework might provide yet another account for age effects. According to this framework, the youngest age group – in contrast to the two older two age groups – might represent the to-be-tracked dot in a topological, one-object-unit mode. However, representation of the complete configuration might indeed be helpful when tracking objects (as this might improve the ability to, e.g., calculate trajectories, remember object locations etc.; Allen et al., 2004; Oksama and Hyönä, 2004; Scholl, 2009). Presumably, making use of these abilities is especially helpful when the objects within the display get closer (Dukette and Stiles, 2001). Given the limitations of the spatial representation system available to young children, increased ACC involvement in this group might represent a compensatory mechanism, e.g., by increasing the salience of the single to-be-tracked object which is still represented in a less elaborated spatial mode in order to fulfill the task in a successful manner.

Apart from activation in the insula-cingulate salience network, a number of further areas showed increased precision-related activity especially in the youngest sample. Such activations in additional, i.e., not typically task-related areas in children are common in developmental fMRI studies (e.g., Konrad et al., 2005; Velanova et al., 2008; Rubia et al., 2010) and interpreted either in terms of inefficient or compensatory recruitment forced by the fact that mature networks cannot yet be activated by younger children (e.g., Geier et al., 2009) or as a result of still-changing communication between networks during childhood and adolescence (Gu et al., 2015; Marek et al., 2015). A region that frequently shows such properties is the precuneus (e.g., Scherf et al., 2006; Velanova et al., 2009). It was proposed that additional precuneus recruitment may reflect children’s limited access to other parietal regions responsible for more sophisticated processing in adults (Scherf et al., 2006). Interestingly, in the present study precuneus as well as posterior cingulate gyrus and lateral occipital cortex showed a common developmental pattern characterized by greatest recruitment in 7-year-olds, significantly reduced activation during late childhood, and an intermediate level of activation in adults. Older children thus seem to rely less on these areas than the other two age groups.

### Limitations

The relatively small sample size of the present study may constitute a potential limitation. However, the object tracking paradigm elicited very strong behavioral effects of age group and distance (effect sizes of η^2^ = 0.86 and η^2^ = 0.61, respectively) and for their interaction (η^2^ = 0.53). Also, the activation effects obtained in the present study were strong, particularly the age effects on attentional resolution, which are at the focus of our interest (cf. **Table 4**). Our study, thus, does not seem to suffer from a general problem in identifying attention-related brain activations or developmental age effects. Nevertheless, we cannot exclude that power may be insufficient for observing more subtle effects. Future studies with larger sample sizes will have to resolve this.

## Conclusion

This study demonstrates that general maturation principles apply to central aspects of selective attention, i.e., the ability to track a target with attention and the ability to select targets with increasingly fine-grained attentional precision. Firstly, the core networks supporting both abilities are principally on-line from an early age on. However, secondly, refinements are both observed in quantitative and qualitative terms and can be interpreted as an optimized recruitment of structures needed to perform the task at hand. More generally, the results of this study support an emerging picture of increased use of more widely distributed and functionally specialized networks with development. This neurofunctional change enables the developing observer to perform visuo-attentional tasks with increasing spatial precision.

## Author Contributions

KW, RD, TP, and CF conceived and planned the experiments. KW, EGW, JvdB, SV, and UN performed the experiments. KW, EGW, and CF analyzed the data. KW and CF interpreted the data and wrote the manuscript. EGW, JvdB, and RD critically revised the manuscript. All authors approved the version to be published.

## Conflict of Interest Statement

The authors declare that the research was conducted in the absence of any commercial or financial relationships that could be construed as a potential conflict of interest.

## Footnotes

1 http://www.fil.ion.ucl.ac.uk/spm

2 http://cibsr.stanford.edu/tools/human-brain-project/artrepair-software.html

3 https://afni.nimh.nih.gov/pub/dist/doc/program_help/3dClustSim.html
